# Antidepressant-Like Activity of Naringenin, an Important Agro-Industrial Resource, Complexed with β‑Cyclodextrin

**DOI:** 10.1021/acsomega.5c06553

**Published:** 2026-01-19

**Authors:** Roxana B. A. Teles, Talita L. N. Gonçalves, Abrahão L. B. S. Reis, Fernanda E. Leite, Ana L. M. Souza, Raimundo G. Oliveira-Júnior, Ana P. Oliveira, Edilson B. Alencar-Filho, Fernanda P. R. A. Ribeiro, Luciano A. A. Ribeiro, Lucas M. M. Marques, Lucindo J. Quintans-Júnior, Jullyanna S. S. Quintans, Jackson R. G. S. Almeida

**Affiliations:** † University of Pernambuco (UPE), 56328-900 Petrolina, Pernambuco, Brazil; ‡ Center for Studies and Research of Medicinal Plants (NEPLAME), Federal University of Vale do São Francisco (UNIVASF), 56304-205 Petrolina, Pernambuco, Brazil; § Paris Cité University, Faculty of Health, 75013 Paris, France; ∥ Federal Institute of Sertão Pernambucano (IF Sertão), 56314-520 Petrolina, Pernambuco, Brazil; ⊥ Department of Pharmacy, Federal University of Vale do São Francisco (UNIVASF), 56304-205 Petrolina, Pernambuco, Brazil; # Faculty of Pharmaceutical Sciences of Ribeirão Preto, University of São Paulo, 14040-903 Ribeirão Preto, São Paulo, Brazil; ∇ Department of Physiology, Federal University of Sergipe (UFS), 49100-000 São Cristóvão, Sergipe, Brazil

## Abstract

The objective of this work was to investigate the effect on the central nervous system (CNS) of naringenin (NR) and of the inclusion complex obtained with its incorporation into β-cyclodextrin (NR-βCD) in mice, using experimental models. The possible participation of the monoaminergic system in the antidepressant activity of NR was also studied. NR and NR-βCD, when administered acutely (20 and 40 mg/kg, p.o.), produced a significant reduction in the immobility time (*p* < 0.05), an effect comparable to imipramine (30 mg/kg, p.o.). The reduction in immobility time observed after NR treatment (20 mg/kg, p.o.) was prevented by pretreatment with haloperidol (0.2 mg/kg, i.p., a D_2_ receptor antagonist) and yohimbine (Yob) (1 mg/kg, i.p., an α_2_-adrenoceptor antagonist), but not with ondansetron (1 mg/kg, i.p., a 5-HT3 receptor antagonist). In summary, NR and NR-βCD produced an antidepressant-like effect with no change in locomotor activity. Furthermore, this anti-immobility effect seems to be mediated by interaction with dopaminergic and noradrenergic systems.

## Graphical abstract

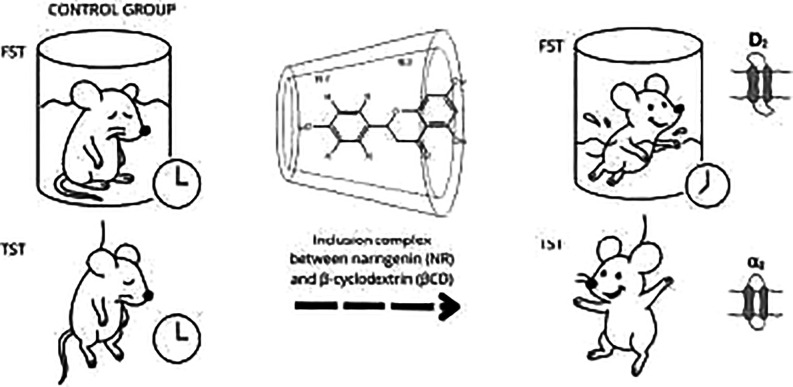

## Introduction

Depressive disorders are chronic and multifactorial diseases, which are a leading cause of morbidity and mortality worldwide.^1–3^ Although their pathological mechanisms are not clearly understood, metabolic dysfunctions of neurotransmitters such as noradrenaline (NA), serotonin (5-HT), and dopamine are often observed in patients with depression conditions. Hypothalamic–pituitary–adrenal (HPA) axis dysfunction, oxidative stress, and also neuroinflammatory, neurotrophic, and neuroimmune factors could also be present in their pathological development.^4,5^

Most of the currently available antidepressant drugs act on the monoamine neurotransmission system by inhibition of monoamine reuptake, antagonism of inhibitory presynaptic monoamine receptors, or monoamine oxidase (MAO) inhibition. However, 30% of patients do not respond to pharmacological treatment, even after exhaustive attempts with different therapeutic strategies. Thus, it is essential to discover new antidepressant drugs that can be used as an alternative treatment.^6,7^

The flavonoid naringenin (4′,5,7-trihydroxyflavanone) is a flavanone with neuroprotective activity, mainly related to monoamine oxidase inhibitory activity. Although this flavonoid is practically found in all parts of citrus fruits, only its pulp is used by most of the food and beverage industries, while the rest is discarded, leading to relevant amounts of wasted naringenin (NR). This compound has low solubility in water and poor oral bioavailability.^8^

In recent decades, complexation of bioactive compounds with cyclodextrins (CDs) has been pointed out as an alternative to improve solubility, permeability, and chemical stability.^9^ CDs are natural polymers composed of *D*-glucopyranose units that, together, give rise to cyclic frusto-conical structures. The most common are α, β, and γ-cyclodextrins (α, β, and γ-CDs), containing respectively 6, 7, or 8 units of glucopyranose, with primary and secondary hydroxyl groups distributed on the external surface (hydrophilic), while the internal cavity of the molecule is essentially hydrophobic. Due to these chemical characteristics, CDs are usually employed to host low-solubility compounds, improving their bioavailability and conferring better plasma stability.^10–12^

This work describes for the first time the antidepressant-like effects in vivo of NR and its inclusion complex with β-cyclodextrin (NR-βCD) obtained from a simple and eco-friendly procedure, adding value to the use of this important flavonoid, which is often wasted in industrial processes.

## Results and Discussion

### Characterization of NR-βCD by Scanning Electron Microscopy (SEM)

SEM is a technique used to determine the structural aspects of raw materials such as βCD, phytocompounds, and their inclusion complexes.^13–19^ As shown in Figure 1, naringenin (a) exhibited a needle-like crystal form, whereas βCD (b) showed the structure of large irregular blocks. The physical mixture (PM) (c) revealed some similarities to both isolated compounds, including characteristic naringenin crystals and irregular βCD blocks. However, inclusion complex (d) showed smaller and distinct structures of isolated components or a physical mixture. Together, SEM images compared to literature data^14^ suggest that a new material was obtained.

**Figure 1:**
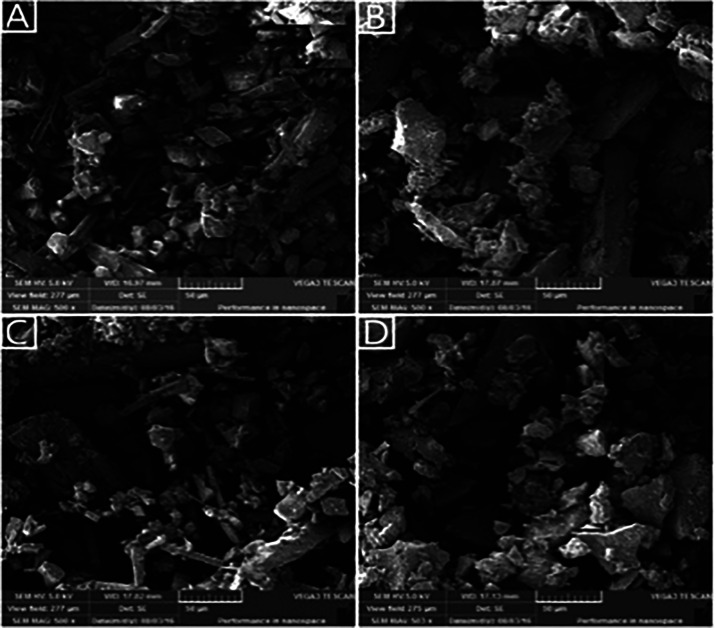
SEM micrographs of (A) naringenin, (B) βCD, (C) naringenin–βCD physical mixture, and (D) naringenin–βCD inclusion complex.

### Fourier Transform Infrared Spectroscopy (FTIR) Analysis

Several alterations were observed in the FTIR spectra of NR-βCD compared to spectral data from the isolated molecules (Figure 2), including (a) band disappearance at 3271 cm^–1^ (O–H, axial deformation) and 2925 and 2835 cm^–1^ (C–H sp^3^, axial deformation) for the guest compound and (b) a band intensity increase at 3303 cm^–1^ (O–H, axial deformation of intermolecular bonds) and the maintenance of the 1024 cm^–1^ stretching (axial deformation of free O–H) for the host molecule.^15^ A band intensity increase at 3303 cm^–1^ indicates a strong host–guest compound interaction, while the 1024 cm^–1^ stretching reinforces the hypothesis that no chemical interaction takes place on the βCD external surface.^15^ These data suggest that the complexation of the guest molecule occurs by the interaction of its A and B rings with the internal cavity of βCD.

**Figure 2:**
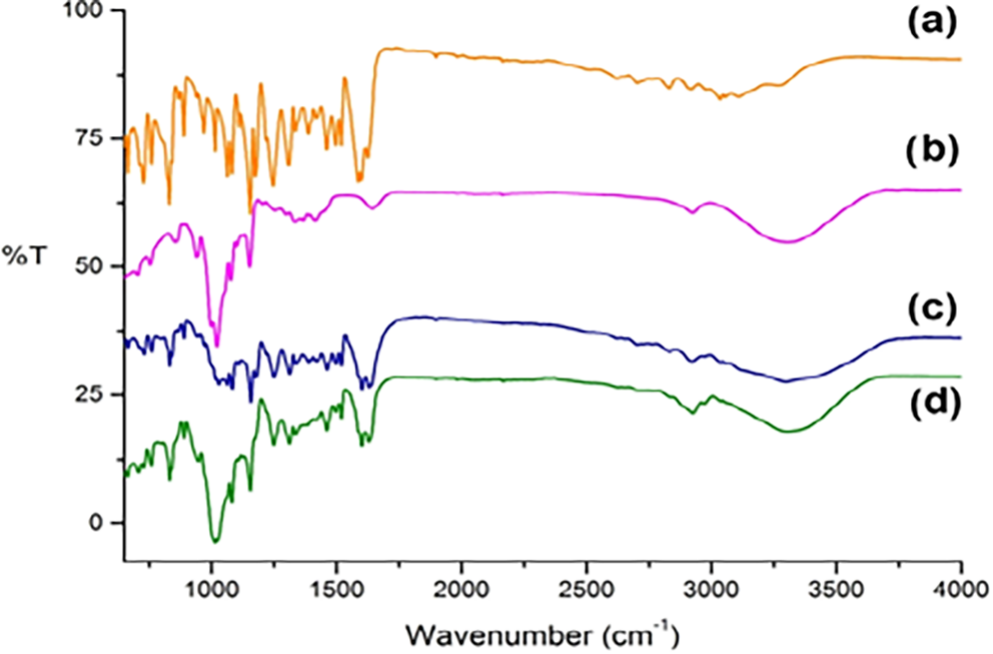
FTIR spectra of (a) naringenin, (b) βCD, (c) naringenin–βCD physical mixture, and (d) naringenin–βCD inclusion complex.

### Nuclear Magnetic Resonance (NMR) Analysis

NMR is one of the most important spectroscopic techniques to provide evidence of the complexation mechanism of host–guest molecules. Alterations in the chemical shifts of the protons H-3 and H-5 of βCD are often used to confirm the formation of inclusion complexes.^13,14,17–19^ In our study, changes in the chemical shifts of βCD were observed after naringenin complexation (Table 1). Apart from H-5, all protons suffered slight but not significant changes in chemical shifts. The highest displacements were observed for H-5 (Δδ = 0.1233 parts per million (ppm)), showing a larger interaction among the internal βCD protons and naringenin, indicating that its inclusion in the host molecule was achieved. A ^1^H–^1^H rotating-frame overhauser spectroscopy (ROESY) experiment was performed to better characterize the mode of interaction between the guest and host molecules.

**Table 1:** ^1^H NMR, Chemical Shifts (δ, ppm) in D_2_O of βCD and Changes Observed for NR-βCD^a^

proton	δ_βCD_	δ_NR‑βCD_	Δδ
H-1	4.9805	4.9790	0.0015
H-2	3.5580	3.5344	0.0236
H-3	3.8816	3.9249	0.0433
H-4	3.4977	3.4168	0.0809
H-5	3.7703	3.6470	0.1233
H-6	3.7912	3.7371	0.0541

a Δδ = δβCD – δNR-βCD (positive or negative values). Data obtained from a Bruker Avance III 400 MHz. βCD: β-cyclodextrin; NR-βCD: inclusion complex (1:1) naringenin-β-cyclodextrin.

In ^1^H–^1^H ROESY experiments, nuclear overhauser effect (NOE) interactions among spins separated by distances shorter than 4 Å enable the formation of cross-peaks, providing important information about the structure of the inclusion complex. The transfer of nuclear spin polarization from one spin to another via cross-relaxation allows verification of how the guest molecule enters the host-compound cavity.^13^

As seen in Figure 3, the ^1^H–^1^H ROESY spectrum showed strong correlations between the B-ring aromatic protons (H-2′/H-6′ and H-3′/H-5′ at 6.85 and 7.25 ppm, respectively) from the NR structure and H-5 protons (3.77 ppm) from βCD. These results corroborate literature data^10,20^ and confirm the inclusion complex formation by the entry of the NR C-ring into the βCD internal cavity, as previously pointed out by FTIR analysis.

**Figure 3:**
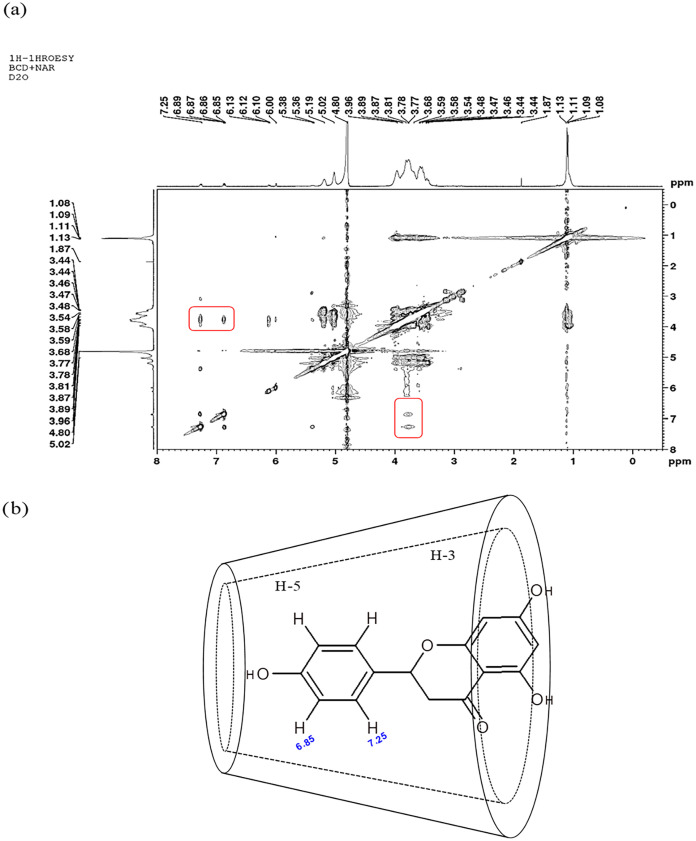
Rotation overhauser effect (ROESY) of the NR-βCD inclusion complex in D_2_O at 289 K (a). Possible interaction mode between naringenin and β-cyclodextrin (b).

### Molecular Docking Results

Figure 4a shows the 10 best conformations after docking results, clustered by Autodock according to the binding energy and root mean square deviation (RMSD) values. Naringenin presents a preferential volume of occupancy in the core of the βCD carrier, denoted by the proximity of the best conformations. Energy values and RMSD for each conformer are listed in Table 2. The energies obtained after the semiempirical calculation show some inversions in relation to the sequence obtained by Autodock. However, the proximity between the geometries (Figure 4 and RMSD values) allows us to conclude that the insertion mode presented through the flavonoid B-ring is the most realistic solution (Figure 4b), in light of ROESY data.

**Figure 4:**
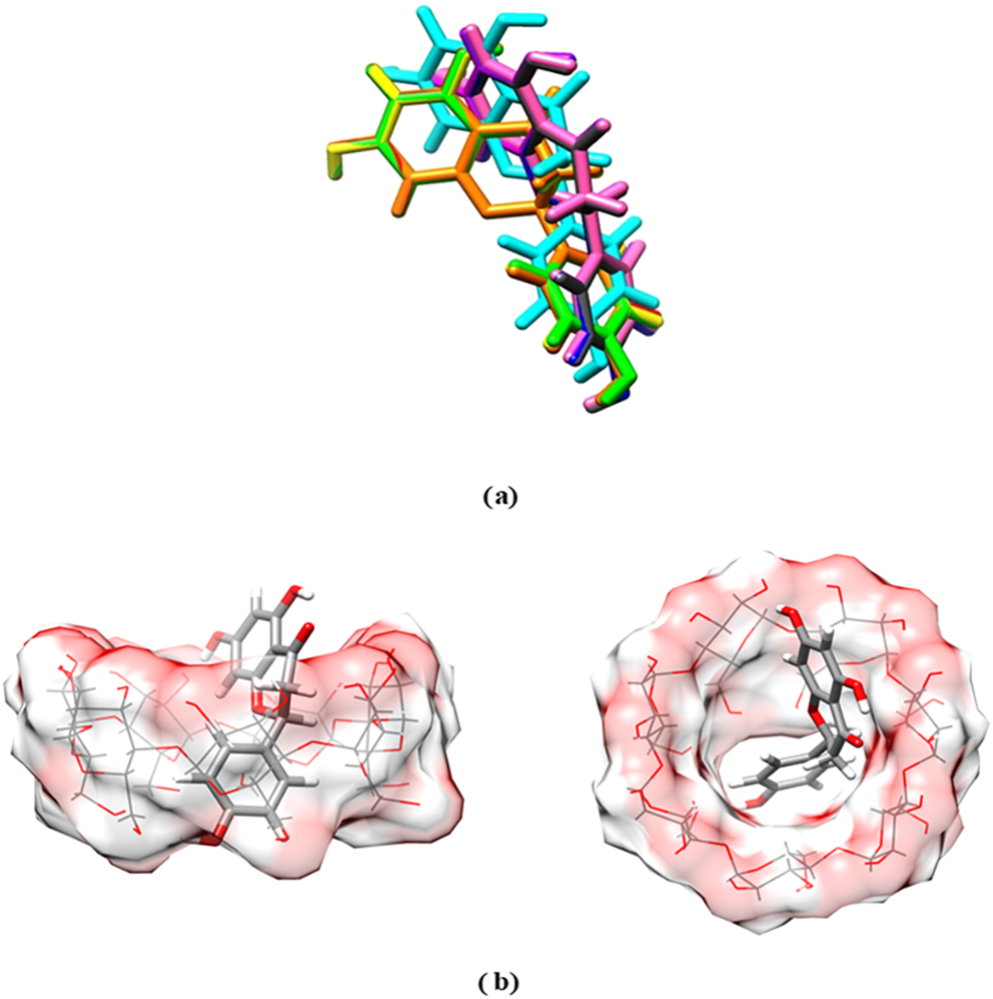
Ten best conformations of naringenin after molecular docking, clustered by Autodock energy and RMSD values (a). Best docking conformation for NR-βCD after PM6-DH2 in two visualization modes (the surfaces show the βCD based on the atomic van der Waals radii) (b).

**Table 2:** Binding Energies (kcal/mol) and RMSD Values for the 10 Best Conformations of NG in βCD

pose	RMSD	binding energy (Autodock)	binding energy (PM6-DH2)
1	0.00	–5.99	–25.63246
2	0.03	–5.99	–25.64663
3	0.09	–5.95	–25.64663
4	0.11	–5.92	–22.73574
5	1.70	–5.85	–28.06904
6	1.69	–5.85	–29.29503
7	1.68	–5.85	–28.06486
8	1.72	–5.84	–28.05079
9	1.74	–5.84	–29.12899
10	1.45	–5.83	–24.91261

Therefore, the docking analysis confirms the complexation mechanism involving the NR B-ring and internal hydrogens from βCD, as first demonstrated by ^1^H–^1^H ROESY NMR. Yang et al.^15^ showed that both water solubility and thermal stability of naringenin were increased in the inclusion complex with cyclodextrins. In view of the limitations of NR application, we describe next the pharmacological profile of NR-βCD in behavioral models to assess its antidepressant potential.

### Acute Toxicity

NR acute toxicity was evaluated using the Organization for Economic Co-operation and Development (OECD) protocol. According to this protocol, NR was classified in the fifth category (substances with LD_50_ between 2000 and 5000 mg/kg); therefore, it is considered a low toxicity drug. Acute treatments induced no relevant changes in behavior parameters. Organs from treated animals did not present any significant relative weight difference nor relevant macroscopic alteration compared to the untreated group. After 14 days, significant changes in body weight were not observed, and no deaths were recorded, indicating that treatments were not capable of promoting important acute toxicity. These results are in accordance with the results reported by Ortiz-Andrade et al.^21^

### Effects of NR and NR/βCD in the Open Field Test (OFT)

OFT has been described as a well-established paradigm to measure locomotion and exploratory activity in animals when confronted with a stressful or threatening situation.^20^ In this experimental model, NR (20 mg/kg) and diazepam reduced the immobility time in comparison with the control group (*p* < 0.05). NR (40 mg/kg) and diazepam significantly reduced the number of crossings, indicating that both drugs affect exploratory activity. However, when treated with NR-βCD, animals showed no significant changes in these behavioral parameters. Additionally, NR-βCD (40 mg/kg) increased the number of rearings, suggesting a possible central nervous system (CNS) stimulating effect (Figure 5).

**Figure 5:**
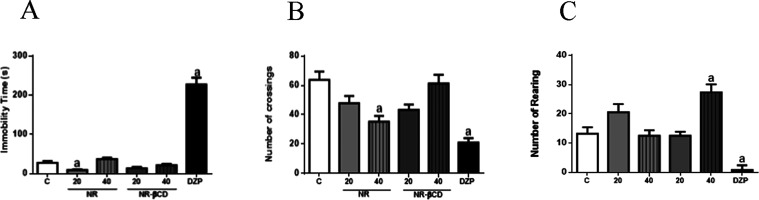
Effects of NR (20 and 40 mg/kg, p.o.), NR-βCD (20 and 40 mg/kg, p.o.), and diazepam (1 mg/kg, i.p.) treatment on (A) immobility time, (B) the number of total squares crossed, and (C) the number of rearing in the OFT. Results are shown as mean ± SEM (*n* = 6) by vertical bars, where ^a^ *p* < 0.05 (compared with the control group) by one-way analysis of variance (ANOVA), followed by post-hoc Tukey’s test.

### Influences of NR and NR/βCD on Depressive-Like Behavior in Mice

To investigate antidepressant-like effects, mice were treated orally with NR and its complex and then evaluated by the tail suspension test (TST) and forced swimming test (FST). According to Figure 6, NR (40 mg/kg) and NR-βCD (20 and 40 mg/kg) significantly decreased the immobility time from 118.2 ± 6.55 s (control group) to 63.83 ± 4.81 s (*p* < 0.05), 68.0 ± 6.84 s (*p* < 0.05), 65.5 ± 6.82 s (*p* < 0.05), respectively, in the tail suspension test. As expected, imipramine also showed significant antidepressant-like activity.

**Figure 6:**
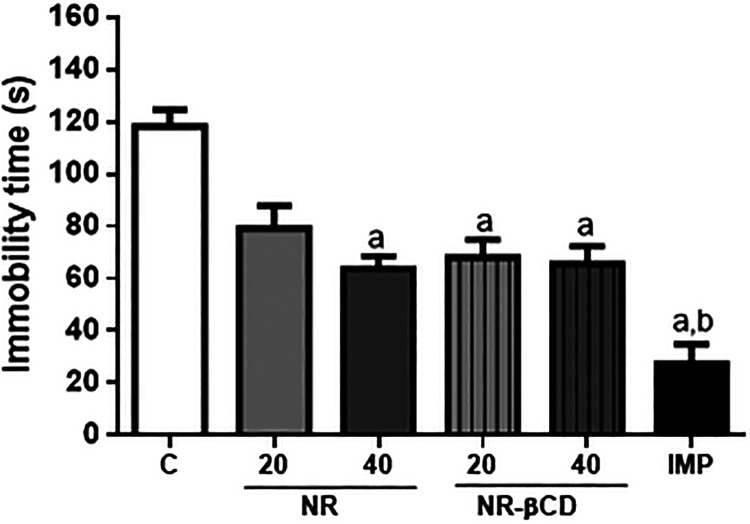
Effects of NR (20 and 40 mg/kg, p.o.), NR-βCD (20 and 40 mg/kg, p.o.), and imipramine (30 mg/kg, i.p.) in the tail suspension test. Values are expressed as mean ± SEM (*n* = 6). ^a^ *p* < 0.05, compared to the negative control group; ^b^ *p* < 0.05, compared to the NR 20 mg/kg by ANOVA followed by Tukey’s test.

As shown in Figure 7, all treated groups have shown a significant decrease (*p* < 0.05) in the immobility time compared to the control group during FST. The antidepressant-like response observed for NR and NR-βCD were more pronounced at lower doses (20 mg/kg).

**Figure 7:**
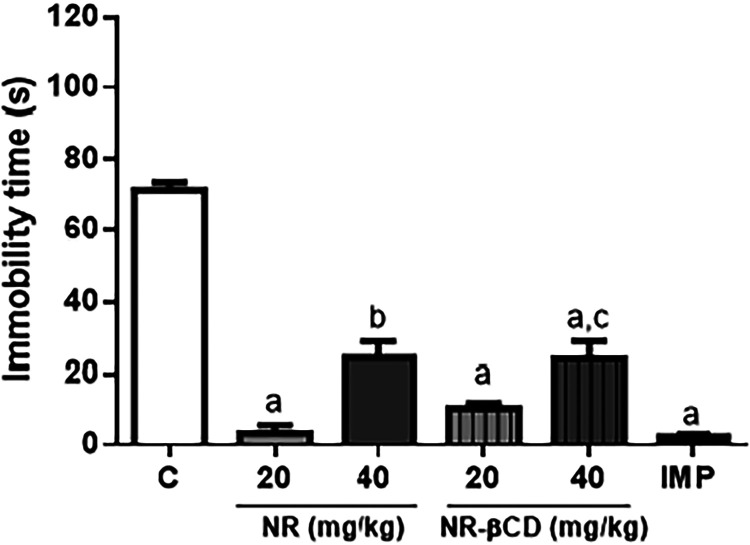
Effects of NR (20 and 40 mg/kg, p.o.), NR-βCD (20 and 40 mg/kg, p.o.), and imipramine (30 mg/kg, i.p.) in the forced swimming test. Values are expressed as mean ± SEM (*n* = 6). ^a^ *p* < 0.05 compared to the negative control group; ^b^ *p* < 0.05 NR 20 mg/kg vs NR 40 mg/kg; ^c^ *p* < 0.05 NR-βCD 20 mg/kg vs NR-βCD 40 mg/kg by ANOVA followed by Tukey’s test.

FST and TST have the same observation parameters (immobility time) that measure the survival behavior of the animals. Both tests are widely used in screening new antidepressant drugs. Variability in response to certain antidepressants indicates potentially different substrates and neurochemical pathways that mediate performance.^22–24^ To comprehend the mechanism of action involved in the antidepressant effect of NR, we next describe the influence of pharmacological antagonists on the behavioral parameter evaluated.

### Possible Mechanism of the Antidepressant-Like Effect of NR Involves the Serotonergic System

Statistical analysis revealed a significantly reduction (*p* < 0.05) in the immobility time induced by NR 20 mg/kg (11 ± 2.71 s), NR+OND (20.0 ± 7.52 s), and OND (5.67 ± 2.49 s) compared to the control group, as presented in Figure 8. However, pretreatment with ondansetron (an antagonist of the 5-HT_3_ receptor) had no effect on the immobility time produced by NR in FST, suggesting that there is no participation of 5-HT_3_ receptors in its antidepressant-like effect.

**Figure 8:**
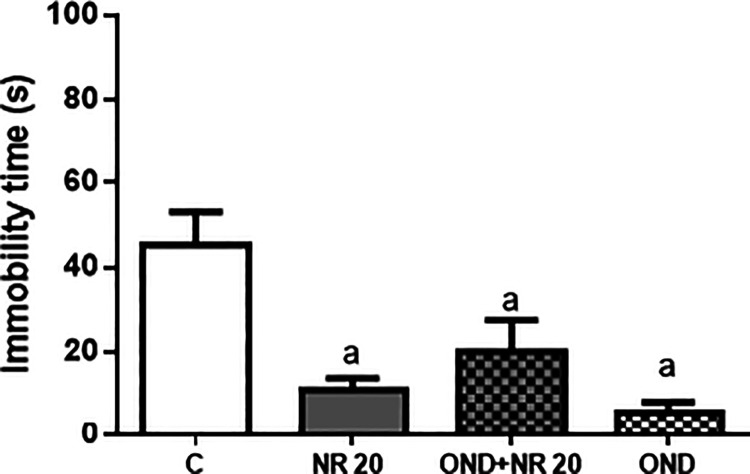
Effect of the pretreatment of mice with ondansetron (1 mg/kg, i.p., a 5-HT_3_ receptor antagonist) on the anti-immobility effect of NR (20 mg/kg) in the FST. Values are expressed as mean ± SEM (*n* = 6). ^a^ *p* < 0.05, compared to the negative control group by ANOVA followed by Tukey’s test.

### Involvement of the Noradrenergic System

As shown in Figure 9, the immobility time in the FST was significantly reduced (*p* < 0.05) in the NR-treated group (11.00 ± 2.78 s) when compared to the control group (45.17 ± 7.85 s). Interestingly, the antidepressant response mediated by NR 20 mg/kg was reversed (46.17 ± 7.36 s) when animals were pretreated with yohimbine (*p* < 0.05), indicating a possible involvement of noradrenergic receptors, especially α-2, in its antidepressant activity.

**Figure 9:**
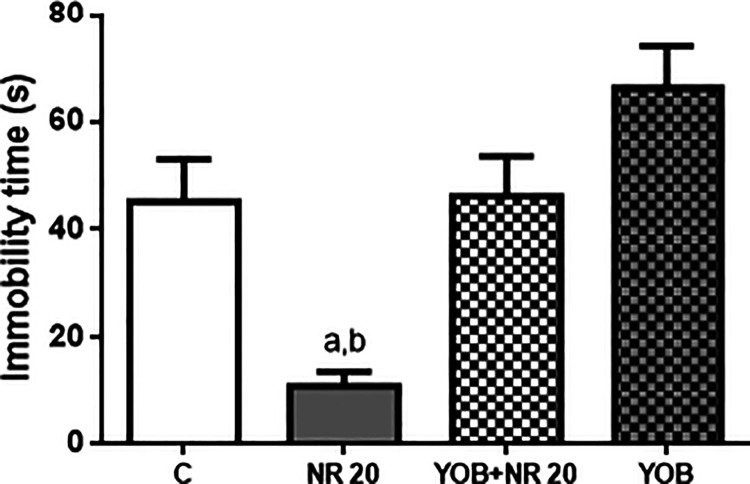
Effect of pretreatment of mice with yohimbine (Yob) (1 mg/kg, i.p.) on the anti-immobility effect of NR (20 mg/kg) in the FST. Values are expressed as mean ± SEM (*n* = 6). ^a^ *p* < 0.05, compared to the negative control group; ^b^ *p* < 0.05, compared to the Yob group 1 mg/kg + NR 20 mg/kg by ANOVA followed by Tukey’s test.

### Involvement of the Dopaminergic System

As expected, immobility time in the FST was significantly reduced (*p* < 0.05) in the NR-treated group (11.00 ± 2.90 s) when compared to the control group (86.33 ± 14.59 s). When the NR-treated group was pretreated with haloperidol (an antagonist of dopamine receptors, especially D_2_ receptors), the NR antidepressant-like effect was abolished, suggesting that the dopaminergic system is probably involved in its pharmacological response (Figure 10).

**Figure 10:**
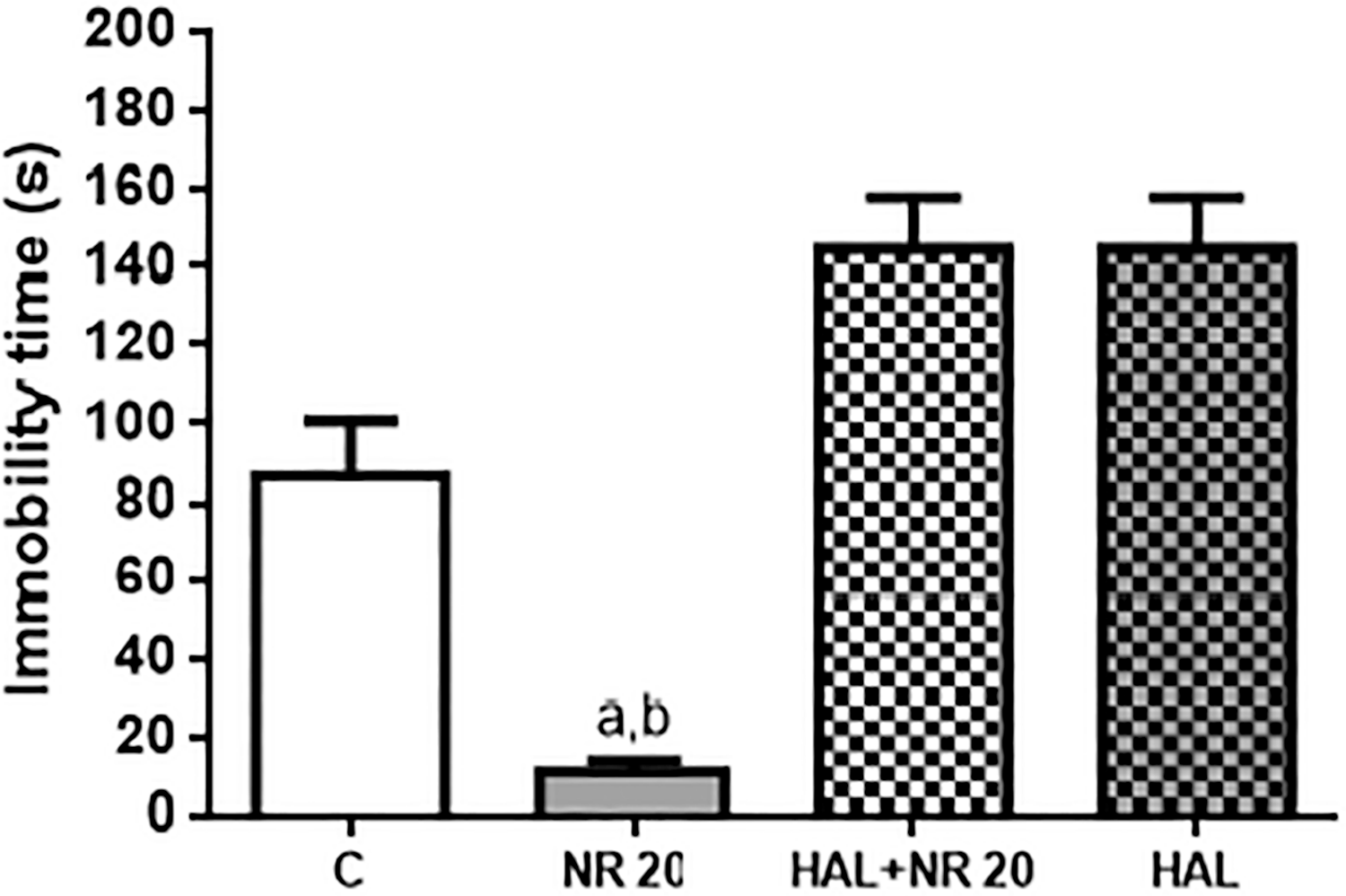
Effect of pretreatment of mice with haloperidol (0,2 mg/kg, i.p., a dopaminergic D_2_ receptor antagonist) on the anti-immobility effect of NR (20 mg/kg, p.o.) in the FST. Values are expressed as mean ± SEM (*n* = 6). ^a^ *p* < 0.05, compared to the negative control group; ^b^ *p* < 0.05, com*p*ared to HAL 0.2 mg/kg + NR 20 mg/kg.

In this work, we describe the effects of acute treatment with naringenin and its complex with β-cyclodextrin after exposure to inescapable stressors or behavioral despair, such as tail suspension and forced swim tests. These animal models have been widely used to study the neurobiology of depression. Naringenin, a high-value-added citrus flavonoid with several biological activities, has emerged as a potential therapeutic agent for the management of a variety of diseases. However, this compound possesses low water solubility and low bioavailability.^17^

Inclusion complexes with βCD have been successfully employed to improve solubility, chemical stability, and bioavailability of poorly soluble compounds.^14^ A range of analytical techniques can be used to confirm the formation of βCD complexes, such as scanning infrared spectroscopy (FTIR), ultraviolet–visible spectroscopy (UV–vis), electron microscopy (SEM), thermogravimetry (TG), differential scanning calorimetry (DSC), X-ray diffractometry (XRD), and nuclear magnetic resonance spectroscopy (NMR).^13,14,16–19^ The combination of the information generated by all of these techniques can prove whether βCD complexes occurred or not. In our study, the inclusion complex between naringenin and βCD was monitored by SEM, FTIR,^1^H NMR, ^1^H–^1^H ROESY NMR, and molecular docking studies. After NR-βCD characterization and toxicity evaluation, the antidepressant effect was assessed by two experimental models (TST and FST). Our results demonstrated that NR and NR-βCD were able to significantly induce behaviors typical of antidepressant-like effects in both tests. Several studies demonstrated that naringenin has different biological activities, such as antioxidant, anti-inflammatory, anticancer, and antidiabetic.^19^ Neuroprotective properties of NR in animal models have also been reported. Its ability to cross the blood–brain barrier (BBB) and exert a wide range of neuronal effects by modulating protein kinase C (PKC) signaling pathways is well-known and makes it a relevant subject of research in the neuropharmacology field.^25–27^

Depression is a disease whose cause is not yet fully understood. The main evidence points to monoaminergic deficiency, reduced γ-aminobutyric acid (GABA) function, dysfunction of the dopaminergic and serotoninergic systems, neurotrophins, and brain-derived neurotrophic factor (BDNF) resulting in loss of neuronal plasticity.^28–30^ In order to elucidate possible mechanisms of action involved in the antidepressant-like activity of NR, we investigated the involvement of serotonergic, noradrenergic, and dopaminergic systems in the FST.

Based on the shown data, 5-HT_3_ does not play any role in the antidepressant-like effect induced by NR. Ondansetron, a selective antagonist of 5-HT_3_ receptors, was ineffective in reversing the decrease in immobility time caused by NR in the FST. Although they are less explored than 5-HT_1a_ and 5-HT_*2*a/2c_, modulation of 5-HT_3_ receptors may contribute to the mechanism of action of antidepressants.^31^ In the brain, 5-HT_3_ receptors control dopamine, acetylcholine release, and the GABAergic system, and their excitation also stimulate cortical GABAergic neurons. Additionally, 5-HT_3_ antagonists are used for postoperative nausea and vomiting treatment, though they have also been suggested to be a possible adjuvant treatment option for obsessive-compulsive disorder.^32,33^ Our results contrast with those obtained by Yi et al.^31^ demonstrated that NR has potent antidepressant-like properties via central serotonergic and noradrenergic systems in TST. Also, naringenin has potent neuroprotective effects by antioxidant mechanisms.^34^

The mechanism of action evaluation revealed that the antidepressant-like effect of NR on FST was blocked by pretreatment with yohimbine, an α_2_-adrenoceptor antagonist, suggesting the involvement of the noradrenergic system in its antidepressant response. Pharmacological evidence of treatment with antidepressants generally implies serotonergic and noradrenergic neurotransmission. However, this latter is still poorly explored and provides an important alternative for depressive disorder management, since presynaptic adrenoceptors regulate the release of other neurotransmitters such as dopamine, serotonin, and GABA.^35,36^

Concerning the investigation of dopaminergic systems, haloperidol (D_2_ receptor antagonist) reversed the reduction in immobility time observed for NR treatment in FST. In previous reports involving Alzheimer’s disease experimental protocols, NR (25–100 μM) considerably decreased Aβ-induced free radical-mediated neurotoxicity in PC12 cells.^35^ In Parkinson’s disease models, strong antioxidant properties were attributed to NR for dopaminergic neurons against oxidative stress caused by *N*-methyl-4-phenyl-1,2,3,6-tetrahydropyridinium hydrochloride (MPP^+^).^36^ Together, previously published data and those reported in this study indicate that NR can modulate the dopaminergic system, attenuating symptoms involved in different CNS conditions, including depressive disorders.

## Conclusions

Naringenin/β-cyclodextrin inclusion complexes have antidepressant-like activity in experimental models. The acute administration of this flavonoid produced an anti-immobility effect in FST and TST, which are widely used tests in the screening of new antidepressant drugs. In addition, this study provides evidence that the antidepressant-like effect of NR in FST is dependent on the interaction with dopaminergic (D_2_ receptors) and noradrenergic (α-2 adrenoceptors) systems. These results encourage the use of NR often wasted in industrial processes for application in the pharmaceutical sector, contributing to the development of new products with antidepressant potential.

## Materials and Methods

### Chemicals

Naringenin (NR, index purity 98%), β-cyclodextrin (βCD, index purity 98%), haloperidol, ondansetron, yohimbine, and diazepam were purchased from Sigma-Aldrich (St. Louis, MO), while imipramine was purchased from EMS (Brazil).

### Preparation of the Inclusion Complex

The inclusion complex (NR-βCD) was obtained by the coevaporation method according to the experimental protocol described by Pinto et al.^12^ The method consisted of the following steps: aliquots corresponding to the rate (1:1 molar ratio) of NR (272 g/mol) and βCD (1135 g/mol) were weighed separately, in an analytical balance. Thereafter, the powders were mixed with 20 mL of distilled water under constant stirring (400 rpm, Quimis Q 261A21, Brazil) for 36 h until complete solvent evaporation. To obtain a physical mixture (PM), NR and βCD were mechanically mixed under ambient conditions in equal molar proportions (1:1 molar ratio). PM and NR-βCD were then characterized by scanning electron microscopy (SEM), Fourier transform infrared spectroscopy (FTIR), and nuclear magnetic resonance (NMR).

### Characterization of NR-βCD

The surface morphology of samples (βCD, NR, PM, and NR-βCD) was examined by using scanning electron microscopy (SEM) in a Tescan-VEGA3 model. For this, powdered samples were mounted into carbon tape attached to an aluminum stub and metallized with gold powder for 250 s and examined using SEM at 5 kV.

### FTIR Analysis

FTIR spectra of βCD, NR, NR-βCD, and PM were obtained in a PerkinElmer Spectrum, Version 10.4.00 using KBr pellets, ranging from 650 to 4000 cm^–1^, and using KBr as spectroscopic blank.

### NMR Analysis

^1^H and ^1^H–^1^H ROESY NMR spectra were recorded for samples (βCD, NR, and NR-βCD) dissolved in D_2_O at 289 K. All experiments were performed in a Bruker Avance III 400 MHz Spectrometer, and the D_2_O resonance at 4.80 ppm was used as an internal reference to report chemical shift values, according to the following equation

Δδ=δ(complex)−δ(free)

Rotating-frame overhauser spectroscopy (ROESY) for the detection of intermolecular nuclear overhauser effects (NOEs) between βCD and NR was conducted for the inclusion complex. The 2D ROESY spectrum was collected with a mixing time of 200 ms under spin lock conditions.^37^

### Molecular Docking Study

Molecular docking procedures^38^ were performed to verify the possible molecular interaction profile between NR and βCD. The crystallographic 3D structure of βCD was taken from the RCSB-PDB database (www.rcsb.org)^39^ (PDB ID: 5MK9).^40^ The complex was edited using the UCSF Chimera program,^41^ by removing the cocrystallized protein and adding the hydrogens to βCD. The ligand was prepared using ACD/Chem Sketch 12.01 software,^41^ followed by an initial geometry optimization at the semiempirical PM3 level through Gauss View 6.0 and Gaussian 09 packages^42,43^ at the CENAPAD cluster environment (http://www.cenapad.ufc.br/). Autodock v4.2, Autodock tools (ADT) v1.5.4 (Autodock, Autogrid, Autotors, Copyright-1991–2000) and MOPAC packages^44,45^ were used to perform the docking algorithms. Initially, Gasteiger charges and polar hydrogens were assigned to the βCD and NR ligand. Nonpolar hydrogens were merged. The ligand was considered flexible on analysis, and the rotatable bonds were chosen automatically by ADT. The affinity maps were calculated into a grid box containing 30, 30, and 30 Å, which involved all the structure of βCD, with an internal spacing of 0.375 Å between the grid points. This box was centered on the Cartesian center of βCD. The Lamarckian Genetic Algorithm (LGA) was used to investigate the most stable conformations of the ligand. The initial population was chosen as 150, with 27,000 as the maximum number of generations. The best value for the maximum number of energy evaluations was 25,000,000 (long). For mutation and crossover rates, 0.02 and 0.8 default values were chosen, respectively, and the elitism rate was 1.0. In the final docking analysis, the best cluster containing conformational similarity, similar binding energy, and little relative values of RMSD (root mean square deviation) between the geometries were observed. Thus, the lowest energy conformations of the most populated cluster were analyzed and considered the most trustworthy solutions. These conformations were submitted to a semiempirical calculation at the PM6–DH2 level using MOPAC software, refining the geometries of the complexes and obtaining more accurate data on the host–guest interaction energy, estimated by the heat of formations Δ*E*_bind_ = Δ*H*°_f_(complex) – (Δ*H*°_f_(ligand) + Δ*H*°_f_(BCD)). The final goals of the docking results were discussed in light of other experimental data.

### Pharmacological Evaluation

All experiments were conducted using 6–8-week-old male Swiss mice (*Mus musculus*) (30–35 g). Animals were randomly separated in groups of six (*n* = 6) mice each in polypropylene cages at a temperature of 22 ± 1 °C and a relative humidity of 60–80% with a light/dark cycle of 12:12 h (7:00 AM/7:00 PM) and free access to food (Purina Labina) and water. Animals were allowed to have a period of acclimation (24 h) before the pharmacological test and were deprived of food but given free access to water 4 h before pharmacological experiments. All efforts were made to minimize animal suffering and to reduce the number of animals in the experiments. This study was performed in accordance with the Conselho Nacional para o Controle de Experimentação Animal (CONCEA, Brazil). Experimental protocols (number 0004/221015) were approved by the Animal Care and Use Committee of the Federal University of Vale do São Francisco (CEUA-UNIVASF, Brazil).

### Acute Toxicity

Oral acute toxicity was assessed according to the Organization for Economic Co-operation and Development (OECD) Guide 423.^46^ Female Swiss albino (*M. musculus*) mice were divided into two groups containing three animals each (*n* = 3), kept in their cages for 5 days for acclimatization to laboratory conditions, and fasted (water ad libitum only) for 3–4 h. After the fasting period, the animals were weighed and treated. The control group received only vehicle (saline solution) orally (gavage), and the other groups received NR or NR-βCD. A high dose of 2000 mg/kg was tested, based on the protocol proposed by the OECD.^46^ Behavioral, motor, and sensory functions were evaluated 60 min after treatments. Animals were individually observed in 30 min, 1-, 2-, 3-, and 4-h periods and daily during the following 14 days.^47^ Mice were also evaluated for potential neurotoxic effects through an open field test (exploratory and locomotor activity) and then in the elevated plus-maze apparatus for observation of an eventual anxiolytic effect. The weight of the animals was evaluated weekly (days 0, 7, and 14), and water and feed intake was recorded daily. At the end of the 14th-day evaluation, animals were anesthetized and euthanized, and the organs were removed for macroscopic analysis.

### Central Nervous System Effects

Behavioral assessments were carried out for neurobehavioral phenotypes, representing spontaneous motor activity (open field test) and antidepressant-like behavior (forced swim test and tail suspension test). Mice were randomly distributed into six groups of 6 animals each (*n* = 6). All behavioral tests were conducted between 10:00 AM and 16:00 PM and scored by an observer blinded to treatment.

### Open Field Test (OFT)

OFT was used to evaluate the exploratory activity. The apparatus consisted of a circular arena with 50 cm in height × 60 cm in diameter, with the base divided into 12 circumcenter quadrants (Insight, Brazil). Sixty min after treatments with saline (control group, p.o.), NR (20, 40 mg/kg, p.o.), and NR-βCD (20, 40 mg/kg, p.o.) and 30 min after treatment with diazepam (1.0 mg/kg, i.p.), animals were individually placed in the central square of the open field and observed for 5 min to record locomotor activity (number of crossings), immobility time, and exploratory activity (expressed by number of rearing and grooming behaviors).^48^

### Forced Swimming Test (FST)

For this experimental model, a glass cylinder containing 11 cm of water at a temperature ranging from 23 to 26 °C (height, 20 cm; internal diameter, 15 cm) was used.^49^ An initial swimming training was conducted in a 15 min session. After 24 h, 6 min test sessions were conducted using animals treated with saline (control group, p.o.), NR (20 and 40 mg/kg p.o.), NR-βCD (20 and 40 mg/kg p.o.), or imipramine (30 mg/kg i.p.). After 30 min (for imipramine) or 60 min (for all other treatments), immobility time was observed during the final 4 min. This behavior was defined as the time spent by the mice floating in the water without struggling and making only those movements necessary to keep their head above the water.

### Tail Suspension Test (TST)

For this test, a manufactured tail suspension box was used (55 cm height × 60 cm width × 11.5 cm depth). To prevent animals from observing the others or interacting with each other, each mouse was suspended within its own three-walled rectangular compartment. Mice were suspended by the tail with a clamp (in the middle of this compartment, and the width and depth were sufficiently sized so that the animal could not touch the walls). The approximate distance between the animal’s nose and the apparatus floor was 20–25 cm. Animals were treated with saline (control group, p.o.), NR (20 and 40 mg/kg p.o.), NR-βCD (20 and 40 mg/kg p.o.), or imipramine (30 mg/kg i.p.). After 30 (for imipramine) or 60 min (for all other treatments), mice were suspended for 6 min, and immobility time was recorded during the final 4 min interval.^50^

### Mechanisms of Action Assessment

To investigate the possible involvement of the serotonergic system (5-HT3 receptors) in the antidepressant-like effect of NR, animals were pretreated with ondansetron (1 mg/kg i.p., a 5-HT3 receptor antagonist). After 30 min, they received NR or vehicles and were submitted to FST. To assess the noradrenergic system involvement, animals were pretreated with yohimbine (1 mg/kg i.p., an α_2_-adrenoceptor antagonist). After 30 min, they received NR or vehicles, and then FST was performed. Furthermore, the role of the dopaminergic system was investigated in animals pretreated with haloperidol, a D_2_ receptor antagonist. Doses used in this study were in accordance with previous reports.^51–54^

### Statistical Analysis

Results were presented as mean ± standard error of the mean (SEM), and statistical analysis was performed using one-way analysis of variance (ANOVA) followed by Tukey’s test. Differences were considered significant when *p* < 0.05. All analyses were performed using GraphPad Prism 6.0 (GraphPad Prism Software, Inc., San Diego, CA).

## Abbreviations

5-HT: serotonin
ADT: Autodock tools
ANOVA: one-way analysis of variance
βCDs: β-cyclodextrin
BBB: blood–brain barrier
BDNF: brain-derived neurotrophic factor
CDs: cyclodextrins
CNS: central nervous system
D_2_O: deuterium oxide
DSC: differential scanning calorimetry
FST: forced swimming test
FTIR: Fourier transform infrared spectroscopy
GABA: γ-aminobutyric acid
HPA: hypothalamic–pituitary–adrenal
i.p.: intraperitoneal
LGA: Lamarckian Genetic Algorithm
MAO: monoamine oxidase
MPP+: *N*-methyl-4-phenyl-1,2,3,6-tetrahydropyridinium hydrochloride
NA: noradrenaline
NMR: nuclear magnetic resonance
NR: naringenin
NR-βCD: naringenin inclusion complex with β-cyclodextrin
NOEs: nuclear overhauser effects
OECD: Organization for Economic Co-operation and Development
OFT: open field test
OND: ondansetron
p.o.: oral
PC12: pheochromocytoma cell line
PKC: protein kinase C
PM: physical mixture
PM6-DH2: parameterized model 6 with dispersion and hydrogen-bonding corrections
ppm: parts per million
ROESY: rotating-frame overhauser spectroscopy
RMSD: root mean square deviation
SEM: scanning electron microscopy
SEM: standard error of the mean
TG: thermogravimetry
TST: tail suspension test
UV–vis: ultraviolet–visible spectroscopy
XRD: X-ray diffractometry
Yob: yohimbine
α-CDs: α-cyclodextrin
γ-CDs: γ-cyclodextrin
